# Apigenin Alleviates Autistic-like Stereotyped Repetitive Behaviors and Mitigates Brain Oxidative Stress in Mice

**DOI:** 10.3390/ph17040482

**Published:** 2024-04-09

**Authors:** Petrilla Jayaprakash, Dmytro Isaev, Keun-Hang Susan Yang, Rami Beiram, Murat Oz, Bassem Sadek

**Affiliations:** 1 Department of Pharmacology & Therapeutics, College of Medicine and Health Sciences, United Arab Emirates University, Al Ain P.O. Box 15551, United Arab Emiratesrbeiram@uaeu.ac.ae (R.B.); 2 Zayed Bin Sultan Center for Health Sciences, United Arab Emirates University, Al Ain P.O. Box 15551, United Arab Emirates; 3 Department of Cellular Membranology, Bogomoletz Institute of Physiology, 01024 Kiev, Ukraine; dmytro.isaev@gmail.com; 4 Department of Biological Sciences, Schmid College of Science and Technology, Chapman University, One University Drive, Orange, CA 92866, USA; kyang@chapman.edu; 5 Department of Pharmacology and Therapeutics, College of Pharmacy, Kuwait University, Safat 13110, Kuwait

**Keywords:** autism spectrum disorder, apigenin, positive allosteric modulator, social features, hippocampal brain slices, electrophysiology, nicotinic α7-nACh receptors, oxidative stress, BTBR mice

## Abstract

Studying the involvement of nicotinic acetylcholine receptors (nAChRs), specifically α7-nAChRs, in neuropsychiatric brain disorders such as autism spectrum disorder (ASD) has gained a growing interest. The flavonoid apigenin (APG) has been confirmed in its pharmacological action as a positive allosteric modulator of α7-nAChRs. However, there is no research describing the pharmacological potential of APG in ASD. The aim of this study was to evaluate the effects of the subchronic systemic treatment of APG (10–30 mg/kg) on ASD-like repetitive and compulsive-like behaviors and oxidative stress status in the hippocampus and cerebellum in BTBR mice, utilizing the reference drug aripiprazole (ARP, 1 mg/kg, i.p.). BTBR mice pretreated with APG (20 mg/kg) or ARP (1 mg/g, i.p.) displayed significant improvements in the marble-burying test (MBT), cotton-shredding test (CST), and self-grooming test (SGT) (all *p* < 0.05). However, a lower dose of APG (10 mg/kg, i.p.) failed to modulate behaviors in the MBT or SGT, but significantly attenuated the increased shredding behaviors in the CST of tested mice. Moreover, APG (10–30 mg/kg, i.p.) and ARP (1 mg/kg) moderated the disturbed levels of oxidative stress by mitigating the levels of catalase (CAT) and superoxide dismutase (SOD) in the hippocampus and cerebellum of treated BTBR mice. In patch clamp studies in hippocampal slices, the potency of choline (a selective agonist of α7-nAChRs) in activating fast inward currents was significantly potentiated following incubation with APG. Moreover, APG markedly potentiated the choline-induced enhancement of spontaneous inhibitory postsynaptic currents. The observed results propose the potential therapeutic use of APG in the management of ASD. However, further preclinical investigations in additional models and different rodent species are still needed to confirm the potential relevance of the therapeutic use of APG in ASD.

## 1. Introduction

The role of the nicotinic acetylcholine (α7-nACh) receptor has attracted growing interest in the search for novel therapeutic modalities for brain disorders including autistic spectrum disorder (ASD). The significant involvement of α7-nAChRs in the neuropathophysiology of cognitive disorders including Alzheimer’s disease [1,2], cognitive deficits [3], schizophrenia [4], autistic spectrum disorder (ASD) [5], and attention deficit hyperactivity disorder (ADHD) [6] is well described. In addition to clinical observations, the contribution of α7-nAChRs to the pathological conditions of ASD has been examined, and the initial results showed an increased number of cholinergic neurons in the basal forebrain of autistic children, as compared to adult patients with smaller and fewer neurons identified [6]. The latter observations suggest a functional dysregulation of cholinergic neurotransmission systems in ASD patients. Moreover, previous preclinical observations showed that the binding of α-bungarotoxin, a marker for α7-containing nAChR subtypes, was significantly higher in the cerebellum [7,8], while α7 immunobinding was clearly decreased in the thalamus of ASD patients [9]. Consequently, the altered cholinergic system in ASD patients appears to precisely include nAChRs, since no alterations were observed at the level of muscarinic receptors and cholinergic synaptic transmission markers including choline acetyltransferase and acetylcholinesterase [7,8,9]. Interestingly, another choline-related metabolite and a positive allosteric modulator of α7-nAChR, namely cotinine, was found to improve the cognitive functions of tested mice using the Fragile X syndrome mouse model, a brain disorder that shares several behavioral deficits and features with ASD [10]. Mccp2-null mice, an animal model applied in preclinical experiments for studying ASD-like features, was shown to exhibit a significant decrease in α7-nAChR-mediated whole-cell currents [11]. Moreover, and in a previous preclinical study, the systemic administration of choline (the selective α7-nAChR agonist) was found to decrease some of the deleterious ASD-like effects on the development of the offspring [12]. Furthermore, transdermal nicotine reduced irritability and improved sleep ratings in an experimental clinical trial, signifying the importance of further research on the role of nAChR agonists and their effects on ASD-like behavioral features [13]. Moreover, the expression levels of CHRNA7 were described to be significantly decreased specifically in the frontal cortex of patients diagnosed with Rett syndrome, a neurodevelopmental and neurodegenerative disease that shares with ASD numerous similar behavioral features [14]. Notably, an increased level of the α7-nAChR antagonist kynurenic acid in the medial prefrontal cortex was found to significantly decrease the brain level of ACh of a BTBR mouse, an idiopathic mouse model that shows core ASD-like phenotypes [15,16,17]. Moreover, and in previous preclinical studies, the systemic pretreatment of BTBR mice with the α7-nAChR agonist, AVL-3288 [18], and nicotine were shown to significantly attenuate the reduced social interaction and repetitive behaviors [3,19]. Accordingly, AVL-3288 was found to act as an allosteric modulator on both nicotinic and GABA_A_ receptor subtypes in the BTBR mouse model, therefore mitigating autism-like behaviors including not only the core symptoms, but also many of the associated comorbidities [18]. Furthermore, and in another preclinical study, other positive allosteric modulators of α7-nAChR were found to provide valuable effects on the sociability behaviors of adolescent rats with prenatally valproic acid-induced ASD [20].

**Figure 1:**
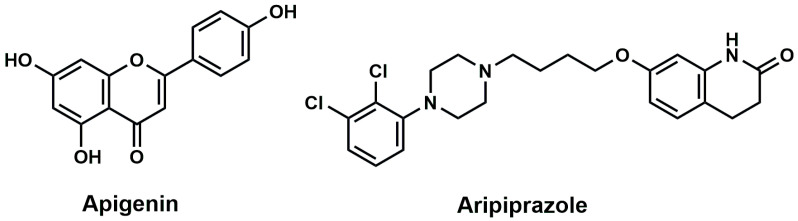
Chemical Structures of Apigenin and aripiprazole.

The potential of flavonoids and flavonoid metabolites in the treatment of neurodegenerative pathology in disorders of cognitive decline is well known and the emerging role of flavonoids in ASD is well documented [21,22]. Observations from preclinical studies in rodents suggested that systemic treatment with flavonoids mitigated oxidative stress parameters, reduced the release of proinflammatory mediators, and promoted pro-neurogenic effects [22]. Moreover, a large body of previous evidence showed that flavonoids ameliorated the core symptoms of ASD, such as social deficits, repetitive behavior, learning and memory impairments, and motor coordination [22]. Interestingly, the natural plant flavonoid apigenin (APG) has been shown to exert several beneficial effects on a wide range of brain disorders. Hence, research over the past two decades clearly showed that APG exhibits, in addition to its neuroprotective effects, anti-inflammatory as well as antioxidant properties [23,24]. Interestingly, and in earlier studies, our research group succeeded in identifying APG as a potent positive allosteric modulator of α7-nAChRs expressed in Xenopus oocytes [25] and mammalian cell lines [26]. Therefore, and as a continuation of our research on APG, the in vitro effects of apigenin on α7-nAChRs in the native neurons of the hippocampus were investigated. To clarify the possible involvement of α7-nAChRs and ACh in the effects observed for APG, the ability of methyllycaconitine (MLA) to reverse the APG-provided effects on hippocampal tissues were assessed. In addition, the in vivo enhancing effects of the systemic administration of APG (10–30 mg/kg) on ASD- and anxiety-like performances of male BTBR mice were assessed in a marble burying test, cotton shredding test, and self-grooming test, applying the reference drug aripiprazole (ARP, 1 mg/kg) (Figure 1). Furthermore, the effects of APG on oxidative stress levels in the hippocampus and cerebellum of treated BRBR mice were evaluated, as BTBR mice have been reported to have elevated levels of oxidative stress with a deficient enzymatic anti-oxidant response that is suggested to be associated with the exaggerated repetitive behavior [27,28].

## 2. Results

### 2.1. APG and ARP Significantly Reduced Burying Behaviors of BTBR Mice in MBT

**Figure 2:**
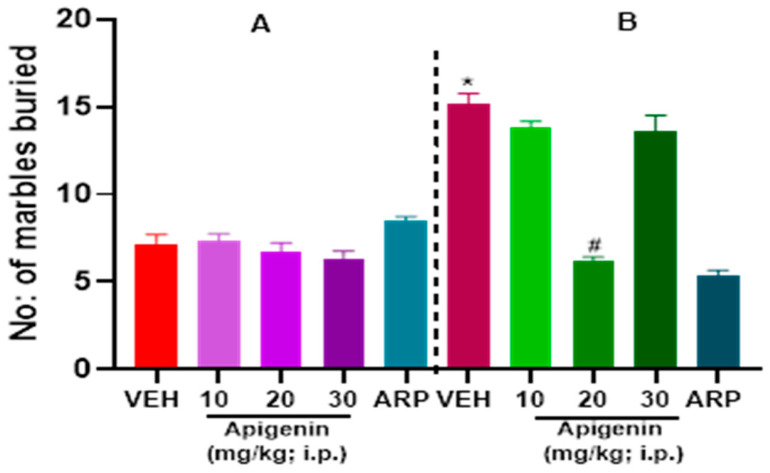
Apigenin alleviated increased marble burying behaviors in BTBR mice. In C57 (**A**) and BTBR (**B**), the number of marbles buried was calculated. When compared to C57 mice, BTBR mice buried noticeably more marbles. Marble burying in C57 mice was not significantly affected by VEH or APG treatment. APG (20 mg/kg, i.p.) and reference drug ARP (1 mg/kg, i.p.) substantially reduced the amount of marble buried in BTBR mice. Data are expressed as the mean ± SEM (n = 6). * *p* < 0.0001 vs. VEH-treated C57 mice. # *p* < 0.001 vs. VEH-treated BTBR mice.

As expected, C57 mice failed to display repetitive behaviors and hence did not execute marble burying capacities during 30-min observation (Figure 2A). Figure 2 illustrates how the marble burying behavior in BTBR and C57 mice was mitigated following systemic treatment with the reference drug aripiprazole (ARP) (1 mg/kg) and APG (10, 20, and 30 mg/kg, i.p.). A two-way ANOVA’s findings suggested that there was a statistically significant impact for treatment [F(4, 47) = 35.11, *p* < 0.0001], strain [F(1, 47) = 88.85, *p* < 0.0001], and the interaction between strain and treatment [F(4, 47) = 50.49, *p* < 0.0001]. Compared to the VEH-treated BTBR mice, there was a significant decrease in the number of marbles buried following the APG (20 mg/kg) treatment of BTBR mice (*p* < 0.0001) (Figure 2B). However, no significant effect was observed following the systemic treatment of BTBR mice with APG (10 mg/kg), and (30 mg/kg), with (*p* = 0.09) and (*p* = 0.17), respectively, and in comparison, to the VEH-treated BTBR group. Moreover, there was no significant effect between the APG (20 mg)-treated group and ARP (1 mg)-treated group, with (*p* = 0.06). Notably, systemic treatment with APG (10–30 mg/kg) had no effect on the overall number of marbles buried in the control C57 mice groups (Figure 2A). The results observed for APG and the reference drug ARP are presented in Figure 2.

### 2.2. APG and ARP Significantly Decreased Cotton Shredding Behaviors of BTBR Mice in CST

**Figure 3:**
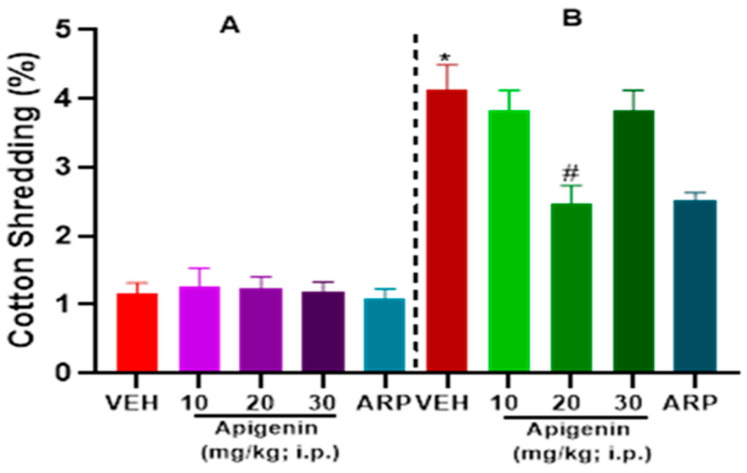
Apigenin reduced typical repetitive behavior in cotton shredding test in BTBR mice. BTBR mice (**B**) showed markedly higher levels of repetitive behaviors when compared to C57 mice (**A**). However, subchronic treatment (21 days) with 20 mg of APG or 1 mg of ARP (i.p.) significantly alleviated the disturbed cotton shredding behaviors of tested BTBR mice. Results shown are mean ± SEM (n  =  6). * *p* < 0.01 vs. VEH-treated C57 mice. # *p* < 0.05 vs. VEH-treated BTBR mice.

As illustrated in Figure 3, VEH-treated BTBR mice (B) showed a significantly increased percentage of cotton shredding behaviors for 30-min evaluation in comparison to VEH-treated control C57 mice (A). A significant effect was observed on strain [F(1, 50) = 219.40, *p* < 0.0001], treatment, [F(4, 50) = 6.289, *p* < 0.05] and strain × treatment interaction [(F(4, 50) = 5.648, *p* < 0.05] as shown following two-way ANOVA analyses. A dose of 10 mg/kg (*p* = 0.52) and 30 mg/kg APG (*p* = 0.52) showed tendency to reduce repetitive cotton shredding behaviors in BTBR mice (Figure 3B). However, only APG (20 mg)-treated BTBR mice had a significant decline in the cotton shredded as compared to VEH-treated BTBR mice (*p* < 0.003) (Figure 3B). Furthermore, the observed effects in BTBR mice with APG (20 mg/kg) were statistically comparable to those of the reference drug ARP (1 mg/kg), since no statistically significant difference was observed between both treated groups (*p* = 0.80). Nevertheless, APG (10–30 mg/kg) showed no effect on the percentage of cotton shredding behaviors in all control groups of C57 mice (Figure 3A).

### 2.3. APG and ARP Reduced Self-Grooming Behaviors of BTBR Mice

**Figure 4:**
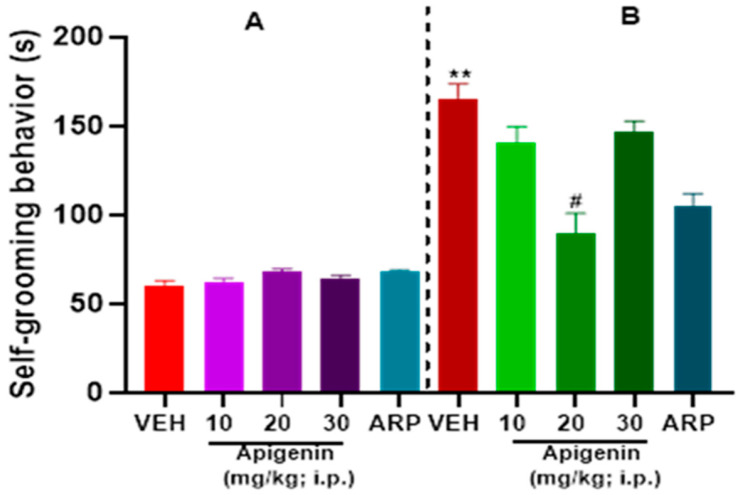
Apigenin decreased spontaneous self-grooming behaviors in BTBR mice. The self-grooming habits of control C57 mice (**A**) and BTBR mice (**B**) were assessed. APG (10, 20, or 30 mg/kg, i.p.), ARP (1 mg/kg, i.p.), or VEH were injected intraperitoneally (i.p.) into each mouse 30 min before the self-grooming behavior testing. When compared to C57 mice, BTBR mice groomed themselves considerably more. In BTBR mice, APG (20 mg/kg, i.p.) and ARP (1 mg/kg, i.p.) substantially reduced the tendency to grooming behaviors. The mean ± SEM time spent grooming each body region (n = 6) was used to express the data. ** *p* < 0.0001 vs. VEH-treated C57 mice. # *p* < 0.005 vs. VEH-treated BTBR mice.

There was a significant difference in the self-grooming behaviors between VEH-treated control C57 mice (A) and VEH-treated BTBR mice (B) as illustrated in Figure 4. Two-way ANOVA analyses showed significant impact on the treatment [F(4, 50) = 29.90, *p* < 0.0001], strain × treatment interaction [F(4, 50) = 33.46, *p* < 0.0001], and strain [F(1, 50) = 129.7, *p* < 0.0001]. Systemic treatment with APG (20 mg/kg, *p* < 0.0003), but not APG (10 mg/kg, *p* = 0.06) or APG (30 mg/kg, *p* = 0.09), significantly lowered the self-grooming period in treated BTBR mice compared to the VEH-treated BTBR group (Figure 4B). Statistical analyses of results showed that there was no significant difference in the self-grooming behaviors between the APG (20 mg)- and reference drug ARP(1 mg)-treated BTBR mice groups (*p* = 0.28). All the treatments with ARP (1 mg/kg) and APG (10–30 mg/kg), however, had no significant effect on the self-grooming behaviors of control C57 mice (Figure 4A).

### 2.4. Effects of APG and ARP on Anxiety Levels and Locomotion of Tested Mice in OFT

Table 1 summarizes the results observed for locomotor activity in both control C57 and BTBR mice. For the total distance travelled, there was a significant effect of strain (*p* < 0.01); however, there was no significant effect observed for treatment or strain x treatment interaction (*p*’s > 0.05) (Table 1). Post hoc analyses of the observed results showed that VEH-treated BTBR exhibited a significantly increased distance travelled when compared with VEH-treated C57 mice (*p* < 0.001). Notably, both mice strains that received APG (10, 20, 30 mg/kg, i.p.) or ARP (1 mg/kg, i.p.) did not show any alterations in the total distance travelled (all *p*’s > 0.05) (Table 1). The latter observations revealed that chronic systemic administration with APG (10, 20, or 30 mg/kg, i.p.) or ARP (1 mg/kg) did not change the time spent in the center of the arena or in the periphery (all *p*’s > 0.05) (Table 1). However, ARP (1 mg/kg, i.p.) significantly alleviated the increased time spent in the center of the arena, with (*p* < 0.05) (Table 1). Interestingly, and for the time spent in the center and in the periphery, there was obviously a significant effect for treatment and the strain x treatment interaction (all *p*’s < 0.01); however, no significant effect of strain (*p* > 0.05) was observed.

### 2.5. APG and ARP Mitigated Disturbed Oxidative Levels in Hippocampus and Cerebellum of Treated BTBR Mice

**Figure 5:**
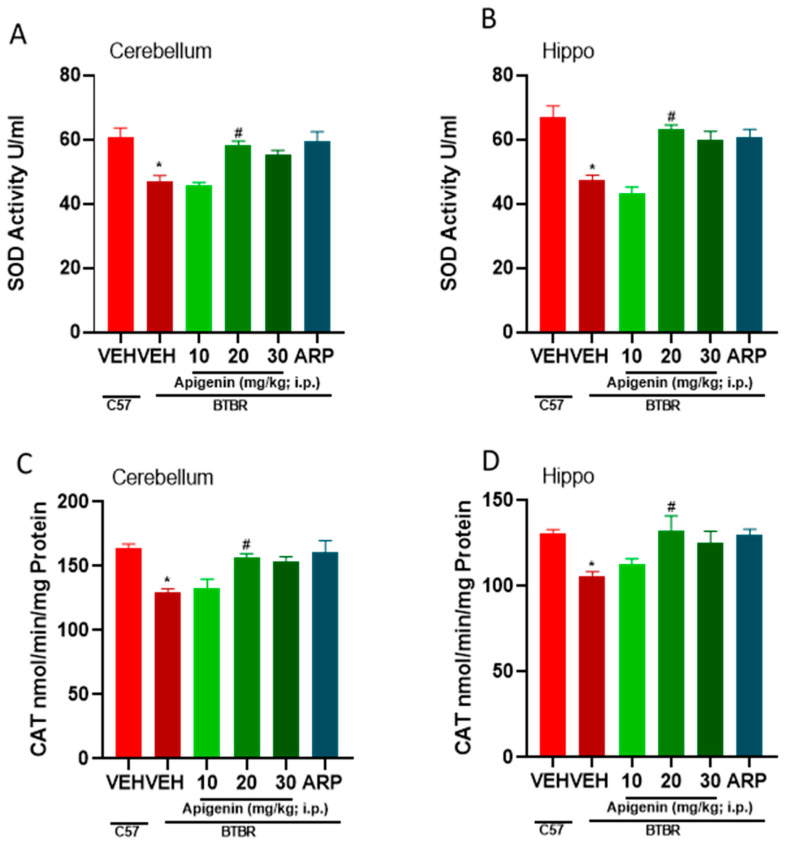
Apigenin mitigated cerebellar and hippocampal oxidative stress levels in brain of pretreated BTBR mice. Enzyme levels of SOD (cerebellum and hippocampus; (**A**,**B**) and CAT (cerebellum and hippocampus; (**C**,**D**) were measured. Results were expressed as the mean ± SEM (n = 5–6). * *p* < 0.005 vs. C57 mice. # *p* < 0.05 vs. VEH-treated BTBR mice. Results are expressed as the mean ± SEM (n = 5–6). * *p* < 0.005 vs. C57 mice. # *p* < 0.05 vs. VEH-treated BTBR mice. And *p* < 0.05 vs. APG (20 mg/kg) BTBR mice. And *p* < 0.005 vs. VEH-treated BTBR mice.

The results showed that VEH-treated BTBR mice had increased oxidative stress levels compared to VEH-treated control C57 mice given the significant drop in antioxidants markers (SOD and CAT) in BTBR mice in the cerebellum and hippocampus, as illustrated in (Figure 5A–D, Table 2). Systemic pretreatment with APG (20 mg/kg) significantly increased the levels of SOD in the cerebellum (*p* < 0.0004) and hippocampus (*p* < 0.0001), compared with VEH-treated BTBR mice (Figure 5A,B). Likewise, the decreased levels of CAT were significantly augmented by APG (20 mg/kg) in the cerebellum (*p* < 0.0001) and hippocampus of treated animals (*p* < 0.01) (Figure 5C,D). Furthermore, the reference drug ARP (1 mg/kg) significantly increased the levels of SOD and CAT in the hippocampus and cerebellum when compared to VEH-treated BTBR mice (Figure 5A–D, Table 2).

### 2.6. Effects of APG on α7-nACh Receptors in the CA1 Region of Stratum Radiatum Interneurons in Mice Hippocampal Brain Slices

**Figure 6:**
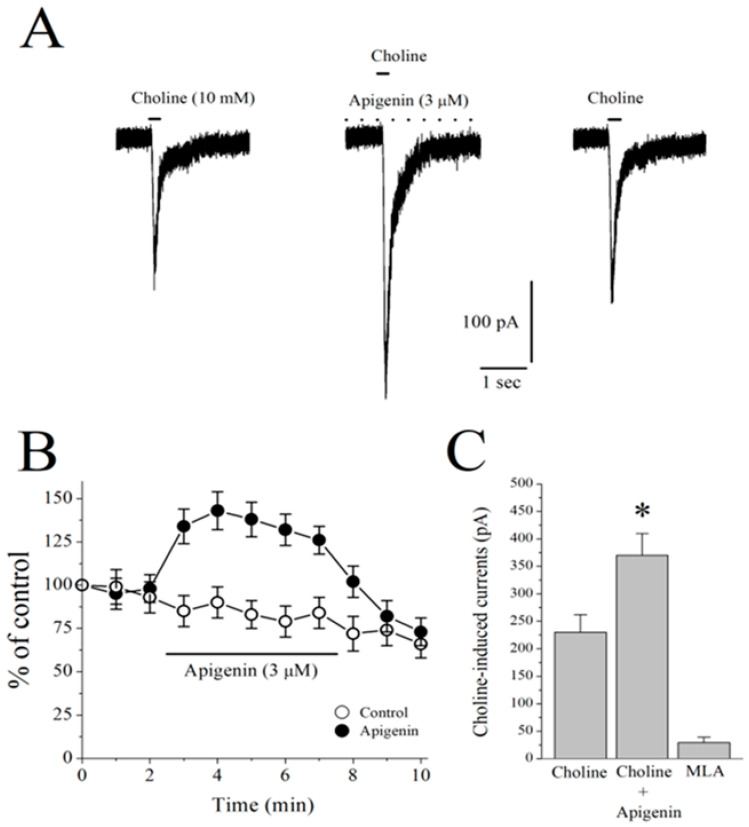
Effects of apigenin on choline-induced ion currents recorded in CA1 area stratum radiatum interneurons of rat hippocampal slices. (**A**) Recordings of choline-induced currents before (control, second panel from left), during (5 min of apigenin), and after (2 min of recovery) the bath application of 3 µM apigenin in hippocampal interneurons. Choline application was represented with a short solid bar on top of the current traces. Dashed line indicates continuing bath application of apigenin. (**B**) Time-course of the effect of vehicle (0.1% DMSO; open circles) and apigenin (3 µM; filled circles) on the peaks of the choline-induced currents. Each data point represents the normalized mean ± S.E.M. of 5 to 7 experiments. Duration of drug application is indicated by the horizontal bar in the figure. (**C**) Summary of the effects of apigenin and methyllycaconitine on the peak amplitudes of choline-induced currents. Bars represent the means ± S.E.M. of 4 to 7 experiments (* *p* < 0.05 vs. control; ANOVA). MLA, methyllycaconitine.

In the whole-cell patch clamp mode, the observed results showed that the focal application of 10 mM of choline, a selective agonist for the α7-nACh receptor, produced rapidly activating and fast desensitizing inward currents that were completely reversed following the bath application of 10 µM of MLA, a selective antagonist for the α7-nACh receptor (n = 5). Moreover, a 5 min bath application of 3 µM of APG significantly potentiated choline-induced currents (Figure 6A). Figure 6B shows the time-courses of obtained effects on the amplitudes of choline-induced currents following APG and the VEH applications. The results showed that APG (3 μM) produced a significant potentiation of the current which was moderately abrogated during a 5 min washout period. A summary of the effect of APG and MLA was presented in Figure 6C.

### 2.7. Effects of APG on Choline-Induced GABA Responses in CA1 Pyramidal Neurons of Mice Hippocampal Slices

**Figure 7:**
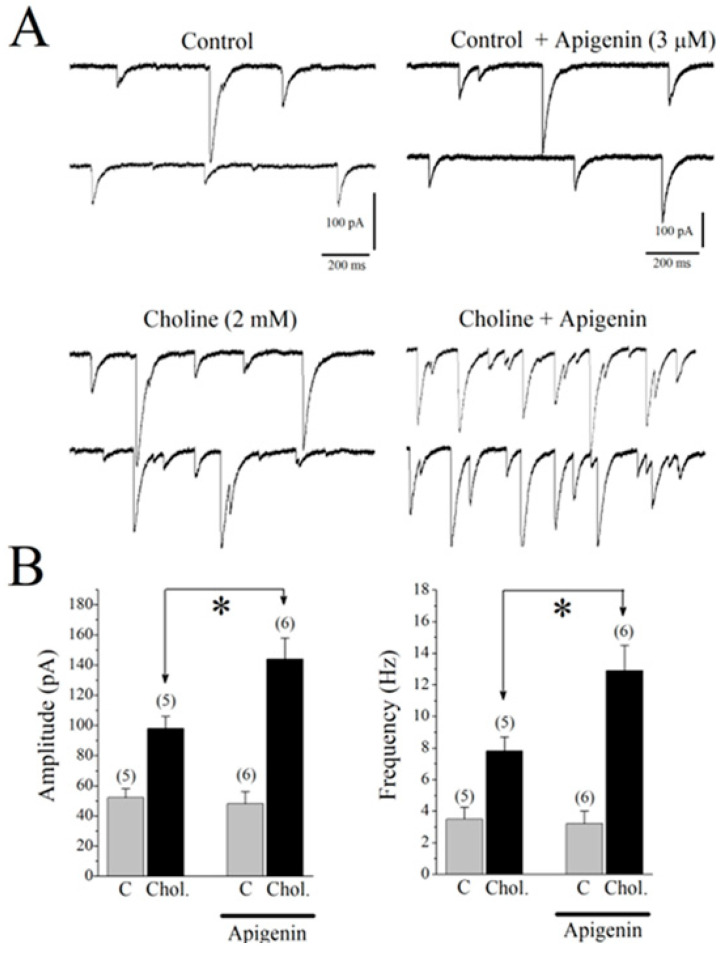
Effects of apigenin on choline-induced enhancement of GABAA receptor-mediated spontaneous synaptic events in CA1 pyramidal neurons. Whole-cell recordings were performed using CsCl-based electrode solution at a holding potential of −70 mV. (**A**) On the left, application of choline (2 mM) for 30 sec increased the amplitudes and frequencies of spontaneous inhibitory postsynaptic currents (sIPSCs; n = 5). On the right, in another cell, choline-induced enhancements of sIPSCs were increased significantly after 2 min preincubation in apigenin (3 µM). (**B**) Summary of the effects of apigenin (3 µM) on choline-induced responses. The averaged amplitudes (on the left) and the frequencies (on the right) of sIPSCs were presented before (control C, gray bars) and after (black bars) choline (Chol. 2 mM) application. For comparison, the effect of choline on the GABAA receptor-mediated sIPSCs is shown in the absence and the presence of apigenin. Bars represent the means ± S.E. of 5 to 6 experiments (* *p* < 0.05 vs. control; ANOVA). C, control; Chol., choline.

The α7-nAChRs are located on both GABAergic and glutamatergic interneurons of the hippocampus, and the application of the selective agonist for α7-nAChRs choline was found to increase both excitatory and inhibitory inputs to CA1 pyramidal neurons [29]. For this reason, we have isolated spontaneously occurring GABAA receptor-mediated synaptic currents (sIPSCs) by pharmacological means (DL-2-amino-5-phosphonovalerate (APV) plus 6,7-dinitroquinoxaline-2,3-dione (DNQX)) and recorded choline-induced GABA responses in CA1 pyramidal neurons of hippocampal slices. Thereafter, the effects of choline were assessed in the absence and presence of 3 µM of APG. The application of choline (2 mM for 30 s) caused a transient increase in the amplitudes and frequencies of sIPSCs that lasted for 1–2 min (Figure 7A,B; n = 6). Choline-induced increases in sIPSCs were completely abolished by a 1 min pre-application of 10 µM of MLA (n = 3), indicating that the effect is mediated by the activation of the α7 nAChR. Two minutes of pretreatment with 3 μM of APG markedly increased the effects of choline on the amplitudes and frequencies of sIPSCs in 7/7 pyramidal neurons (Figure 7B). When miniature IPSCs (mIPSCs) were measured in the presence of 1 µM of TTX, APG (3 μM, 5 min) alone did not change the amplitudes and frequencies of mIPSCs (n = 5). The amplitudes and frequencies of mIPSCs in the presence and absence of APG were 21.4 ± 2.7 pA and 0.9 ± 0.4 Hz (n = 4), and 24.6 ± 3.9 pA and 0.7 ± 0.3 Hz (n = 6), respectively. Notably, the observed results revealed that there were no statistically significant differences between the APG- and VEH-treated groups with respect to the means of amplitudes and frequencies (*p* > 0.05, ANOVA, n = 6), signifying that the test compound APG failed to affect postsynaptic GABAA receptors.

## 3. Discussion

The role of cholinergic dysfunction and the decreased levels of brain ACh in BTBR mice are well documented. Accordingly, and in a recent study in experimental rodents, the dysfunction of cholinergic neurotransmission and the significantly decreased levels of brain ACh were found to be directly associated with ASD-like features of BTBR mice [17]. Consequently, treatment with donepezil, the standard drug of acetylcholine esterase inhibitors, was reported to relieve social memory impairment [30]. Moreover, and in children diagnosed with ASD, the gray and white matter were found to have significantly lower levels of choline, the precursor molecule for the biosynthesis of the ACh neurotransmitter and agonist of α7-nAChRs [31]. Also, significant decreases in the levels of α7-nAChRs were described in several regions of the brain, including the cerebellum, striatum, thalamus, and neocortex of individuals diagnosed with ASD, with the vital aberration being the significant degeneration of cholinergic muscarinic M1 receptors [9,31,32]. In the current study, the alleviating effects observed for the test compound APG (20 mg/kg) on marble burying as well as on the cotton shredding and self-grooming paradigms were statistically similar to those observed for the reference drug ARP (1 mg/kg) (Figure 2, Figure 3 and Figure 4), the only one FDA-approved drug for the clinical use in patients diagnosed with ASD to reduce repetitive behaviors. Our observations are in harmony with several previous studies in which APG has been shown to exhibit positive effects in different rodent models of induced ASD [26,33,34,35,36,37,38]. Notably, and following the previous determination of APG in rat plasma applying the highly selective and sensitive LC-MS/MS method, the calibration curves were found to follow linearity in the concentration range of 0.50–500 ng/mL [39,40,41]. Also, the inter-day and intra-day precisions at different quality control levels within 13.1% and the accuracies ranged from −10.6% to 8.6% [39,40,41]. Moreover, the plasma concentrations of APG following oral and intravenous administration have been studied previously [42,43]. When APG containing plant extracts is given orally at a dose of 8 mL/kg to rats, a maximum serum concentration of 6.7 µg/mL (24.8 µM) was attained [42]. Similarly, oral (5.4 mg) or intravenous (20 mg/kg) administrations of APG preparations have yielded plasma concentrations of 4.5 µg/mL (16.6 µM) and 10.9 µg/mL (40.7 µM), respectively [43]. Since APG is a highly lipophilic compound with a LogP (octanol–water partition coefficient) value of 2.577, its brain concentration is expected to be considerably higher than blood levels [39]. Therefore, the functional modulation of 7-nAChRs demonstrated in this study can be pharmacologically relevant. Moreover, and in a recent preclinical study, the oral administration of APG was able to reverse scopolamine-induced cognitive impairment and neural damage in mice, demonstrating the pharmacokinetic capability of APG to accumulate in the brain of tested mice [44]. Although this is the first study reporting the beneficial effects of APG on ASD-like features in BTBR mice, several flavonoids with some structural similarity to APG have been shown to alleviate ASD-like symptoms in various animal models and clinical studies [21,22]. An additional major objective of the current series of experiments was the assessment of APG capability to mitigate abnormal levels of oxidative stress in two different brain regions of treated BTBR mice, namely the cerebellum and the hippocampus. The observed results showed that BTBR mice with ASD-like paradigms presented significantly decreased hippocampal and cerebellar levels of CAT and SOD of BTBR mice and as compared to control C57 mice (Figure 5, Table 2). However, the systemic administration with APG (20 mg/kg) and ARP (1 mg/kg) showed a significant elevation in the levels of SOD and CAT in the hippocampal as well as cerebellar tissues of treated BTBR mice (Figure 5, Table 2). The latter results on the levels of oxidative stress markers agree with several previous studies in which synthetic ligands with modulating effects on brain ACh were described to show capability of mitigating the oxidative stress levels of assessed animals [45,46]. Notably, the mitigating effects observed for APG (20 mg/kg) on the oxidative stress markers CAT and SOD in the hippocampus as well as cerebellum were similar to those of the reference drug ARP (1 mg/kg). The latter observations explain—at least in part—the mechanism behind the observed enhancing effects of both APG and ARP on the behaviors of tested BTBR mice with ASD-like features in MBT and CST. Furthermore, and following an assessment of APG and reference drug ARP in OFT, ARP was found to completely reestablish the abnormal anxiety levels of assessed BTBR mice (Table 1). The latter observations for ARP are in agreement with several previous studies that demonstrated considerable anxiolytic effects of the reference drug ARP [47,48,49]. However, APG (10–30 mg/kg, i.p.) was not able to provide any alteration on the abnormal anxiety-like behaviors of tested BTBR mice. Also, no behavioral abnormalities were observed that could be linked to any toxic effects of the administered dose range (10–30 mg/kg, i.p.) of APG. In harmony with the latter observations, APG has been reported to induce hepatocyte degeneration and toxicity, mild sedation, and muscle relaxation in murine models at very high doses (100 and 200 mg/kg) [40], a dose range that was not used in our experiments in BTBR and C57 mice. Notably, APG (10, 20, 30 mg/kg, i.p.) and ARP failed to alter the total distance travelled as well as the time spent in the periphery of treated BTBR mice (Table 1). The latter observations at this administered dose range (10–30 mg/kg, i.p.) are crucial to exclude any factors that may confound our positive results in regard to the behavioral improvements provided by APG and/or ARP, such as changes in locomotor activity. Interestingly, our results are in agreement with numerous previous preclinical studies carried out in experimental rodents and in which different types of compounds belonging to the flavonoids, e.g., baicalin, genistein, naringenin, quercetin, hesperetin, catechin, anthrocyanin, epigallocatechin-3-gallate, and luteolin, were able to ameliorate several behavioral ASD-like deficits and mitigate the disturbed levels of oxidative stress in the brains of tested animals, e.g., CAT, MDA, SOD, and GSH [50,51,52,53,54,55,56,57,58,59,60]. Also, our observations agree with the results obtained for APG in a pervious study in which APG provided neuroprotective, anti-amyloidogenic, and neurotrophic effects in an Alzheimer’s disease mouse model, signifying the capability of APG to improve the cognitive functions of tested animals [38].

The observed results indicate that APG potentiates α7-nAChRs in native central nervous system neurons, mitigates disturbed oxidative stress levels, and alleviates ASD-like features in assessed BTBR mice. The results observed in vitro, in vivo, and associated clinical studies strongly propose that dysfunctional α7-nAChRs are involved in the development of ASD and that the stimulation of this subtype of cholinergic receptors has potential valuable effects in the future therapeutic management of ASD. Taking into consideration that various factors could impede the therapeutic applicability of APG, e.g., difficulties linked with its relatively low solubility, poor oral bioavailability, and chemical instability at neutral and slightly alkaline conditions, further evaluations of the therapeutic potential role of APG still must be certified for use in humans, and further research is also needed to help clarify the optimal therapeutic dosage, treatment duration, and the efficacy of APG in neuropsychiatric disorders, e.g., ASD.

## 4. Materials and Methods

### 4.1. Animals

C57BL/6J mice and a strain of BTBR T_ Itpr3tf/J (BTBR) acquired from Jackson Laboratory (Bar Harbor, ME, USA) were used for experiments. The adult males of both mice strains weighed between 28 and 32 g and were 8 weeks old. A temperature-controlled environment, which was between 22 and 25 °C, with a conventional 12 h light/dark was set up to house all the mice. The mice were housed in cages with unlimited access to clean drinking water bottles and a diet of typical rodent chow.

### 4.2. Drugs

The test compound APG, (10–30 mg/kg, i.p.), the reference drug ARP (1 mg/kg, i.p.) hydrochloride, and the CNS-penetrant methyllycaconitine (MLA, 1 µg/kg, i.p.) were obtained from Sigma-Aldrich (St. Louis, MO, USA). The assay kits for superoxide dismutase (SOD) and catalase (CAT) were procured from Cayman Chemical (Ann Arbor, MI, USA).

### 4.3. Experimental Plan

The mice used in this study were C57 and BTBR mice (mice comprising ASD-like behavior) and were given subchronic injections with the test compounds APG and ARP. A vehicle (saline + DMSO) was administered to the control group as VEH-treated BTBR (group 1). Three different doses (10, 20, 30 mg/kg) of APG and a dose of 1 mg/kg for ARP were chosen based on previous studies and were referred to as group 2, 3, 4, and 5. A total number of 6 mice were used for each group, and marble burying, cotton shredding, and self-grooming behavior experiments were performed.

### 4.4. Behavior Tests

#### 4.4.1. Marble Burying Test (MBT)

Repetitive behaviors are commonly found in ASD and pose a serious concern in the individual’s health [61]. As previously described, MBT was carried out in a clean plastic cage with measurements of 10.2 inch × 18.9 inch × 7.9 inch. A habituation period of 10 min was given to each mouse and then carefully removed. Twenty marbles were cautiously positioned atop the bedding (5 columns of 4 marbles each) material and the same mouse was returned. The observer left the room for 30 min, leaving the mice undisturbed. After 30 min of exploration time, the number of marbles buried were counted and then analyzed to compare between the groups. The marbles were counted as buried if 50% or more were concealed under the bedding [45,46,61,62,63,64,65,66].

#### 4.4.2. Cotton Shredding Test (CST)

Following previous published protocols, the initial assessments of the shredding behavior, without pain, were measured during 30-min sessions [45,46,61,62,63,64,65,66]. Each mouse was accustomed to a test cage that was comparable to their home cage devoid of any water or food in order to analyze repetitive behavior. Each mouse in the cage received a cotton/nestlet for 30 min after 5 min of habituation. To determine the percentage of the cotton that was shredded, the cotton was manually weighed prior to and following the test.

#### 4.4.3. Spontaneous Self-Grooming Behavior Test (SGT)

Self-grooming is a recurrently preformed repetitive motor pattern that is an innate complex behavior [65,67]. The spontaneous grooming behavior was calculated in this test as previously described [68]. An individual mouse was positioned in the center of an empty, clear, and clean plastic mouse cage. The measurement for the cage used was 11 inch wide × 6.7 inch long × 4.7 inch high. The entire test was a total of 20 min, in which the first 10 min was given to each mouse as the habituation period. In the next 10 min, grooming of the whole body which includes the body, head, face, nose, paw, and tail was calculated. During the second phase, the observer was seated with a distance of approximately 1.5 m from the test mouse cage.

#### 4.4.4. Open Field Test (OFT)

The effects of APG and reference drug ARP on locomotion and anxiety levels were assessed, following our previously published protocols for the open field test (OFT), by applying a charge-coupled device camera-assisted motion tracking apparatus and software (EthoVision 3.1, Noldus Information Technology, 6709 PA Wageningen, The Netherlands) (Table 1) [46]. During the test sessions, the mice with a higher anxiety degree preferred to move nearer to the walls of the open field box and spent less time in the center, whereas increased time spent in the center designated high exploratory behaviors and lower anxiety levels of the respective tested mouse.

### 4.5. Biochemical Assessments

After perfusion, mice brain regions (hippocampus and cerebellum) were taken and used to perform the enzyme assay kits.

#### 4.5.1. Catalase (CAT) Assay

The CAT Assay Kit from Cayman Chemicals (Ann Arbor, MI, USA) was used to carry out this test. The test uses 4-amino-3-hydrazino-5-mercapto-1,2,4-triazole (Purpald) as the chromogen to quantify formaldehyde, which is produced when CAT reacts with methanol with the help of hydrogen peroxide. Aldehydes and purpald combine to generate a bicyclic heterocycle that, when oxidized, transforms from colorless to purple and can be measured colorimetrically. The manufacturer’s instructions were followed to perform this assay [69]. Each sample’s CAT activity was expressed in nmol/min/mL. CAT activity is calculated as (μM of sample/20 min) X sample dilution. At 25 °C, one unit can be well defined as the quantity of enzyme, per minute, that caused the formation of 1.0 nmol of formaldehyde [70].

#### 4.5.2. Superoxide Dismutase (SOD) Assay

The SOD Assay Kit (Cayman Chemicals, Ann Arbor, MI, USA) was used to carry out this test. Tetrazolium salt is used in the experiment to detect the SOD produced by xanthine oxidase. The assay was conducted in accordance with the manufacturer’s guidelines. One unit is the quantity of enzyme required to catalyze the superoxide radical’s 50% dismutation. This equation was used to determine SOD activity:

SOD activity (U/mL) = {[(sampleLR–y − intercept)/Slope] × (0.23 mL/0.01 mL)} ×  sample dilution

### 4.6. Recordings from Hippocampal Slices

Recordings in the hippocampal horizontal slices (300–350 μm) prepared from 15- to 30-day-old male C57B/6 and BTBR mice using a vibratome (Pelco, Redding, CA, USA) were carried out as described earlier. Whole-cell patches from the somata of CA1 area interneurons were made under visual control using an upright microscope (Nikon Eclipse E600 FN, Garden City, NY, USA) equipped with infrared differential [71,72,73].

### 4.7. Statistical Analyses

For whole-cell patch clamp results, every analysis was performed in duplicate, and statistical evaluation was carried out applying ANOVA by using Origin v8.5 (Microcal Software, Northampton, MA, USA). For the behavioral tests and biochemical assessments, the statistical software PRISM was used to evaluate and graph all of the data. All results were expressed as the mean ± SEM (n = 6). The significance of the relationship between the groups was ascertained using two-way ANOVA (Tukey’s multiple comparisons test). At a 95% (*p* < 0.05) level of confidence, the differences between means were deemed statistically significant.

## 5. Conclusions

Taken together, APG with potentiating effects on α7-nAChRs in native hippocampal neurons showed promising in vivo anti-ASD as well as antioxidative effects in tested BTBR mice. Therefore, the flavonoid APG might address the need for safe and effective drugs with potential to be used in the treatment of neuropsychiatric disorders, e.g., ASD. However, these promising preliminary alleviating effects of APG on ASD-like features still need to be assessed in a battery of additional animal models of ASD to be able to generalize the current observations and conclusions.

## 6. Study Limitations and Future Directions

A limitation of our study is that the docking studies of the test compound APG on several targets including α7-nAChRs are lacking to elaborate with more insights regarding the mechanism of action of APG. Also, further assessments of the therapeutic potential role of APG are still warranted for use in humans, and more research is also needed to enable elucidation on the optimal therapeutic dose range, duration of the treatment, and the efficacy of APG in neuropsychiatric and neurodegenerative diseases, e.g., ASD. Nonetheless, this research question regarding the potential use of APG in neurodevelopmental diseases is interesting and is worth further preclinical as well as clinical research. Collectively, the current findings are valuable for and can encourage future studies to exacerbate the pharmacological effects of the flavonoid APG in different rodent models of ASD.

## Figures and Tables

**Table 1:** Effects of apigenin and aripiprazole on time spent in the center and periphery and the total distance travelled by tested BTBR mice.

Group	Center Time (s)	Periphery Time (s)	Travelled Distance (cm)
VEH (C57)	31.64 ± 3.27	561.63 ± 4.91	2621 ± 197.57
VEH (BTBR)	66.15 ± 4.29 **	536.31 ± 7.27 **	4201 ± 171.23 **
APG (10 mg/kg)/BTBR	61.02 ± 5.63	539.69 ± 11.73	3979 ± 118.98
APG (20 mg/kg)/BTBR	58.971 ± 6.72	542.22 ± 10.28	4036 ± 191.26
APG (30 mg/kg)/BTBR	55.98 ± 6.97	535.92 ± 12.71	3878 ± 352.18
ARP (1 mg/kg)/BTBR	39.84 ± 7.21 ^#^	566.65 ± 9.89	4063 ± 181.09

Data are expressed as the means ± SEM (n = 6). ** *p* < 0.001 vs. C57 mice. # *p* < 0.05 vs. VEH-treated BTBR mice.

**Table 2:** Apigenin and aripiprazole mitigated levels of oxidative stress markers in different brain parts of treated BTBR mice.

	C57 Mice (VEH)	BTBR Mice				
		(VEH)	APG (10 mg/kg)	APG (20 mg/kg)	APG (30 mg/kg)	ARP (1 mg/kg)
**SOD**						
*Hippocampus*	66.4 ± 5.4	49.5 ± 0.5 *	43.36 ± 1.90	54.42 ± 1.54	49.74 ± 1.03	51.8 ± 2.0
*Cerebellum*	60.6 ± 3.1	49.7 ± 1.3 *	45.83 ± 0.84	58.15 ± 1.40 ^#^	51.38 ± 2.13	56.4 ± 1.1
**CAT**						
*Hippocampus*	131.7 ± 3.4	111.4 ± 4.8 *	112.4 ± 3.60	117.7 ± 8.03	110.9 ± 5.41	118.3 ± 6.5
*Cerebellum*	156.7 ± 7.29	130. 7 ± 3.3 *	139.5 ± 8.46	155.2 ± 1.602 ^#^	145.8 ± 3.22	159.2 ± 5.1

Data are expressed as the mean ± SEM (n = 5–6). * *p* < 0.005 vs. C57 mice. # *p* < 0.05 vs. VEH-treated BTBR mice.

## Data Availability

Data are contained within the article.
